# Improving care and wellness in bipolar disorder: origins, evolution and future directions of a collaborative knowledge exchange network

**DOI:** 10.1186/1752-4458-6-16

**Published:** 2012-09-10

**Authors:** Erin E Michalak, Rachelle Hole, James D Livingston, Greg Murray, Sagar V Parikh, Sara Lapsley, Sally McBride

**Affiliations:** 1 Department of Psychiatry, University of British Columbia, 2255 Wesbrook Mall, Vancouver, BC, V6T 2A1, Canada; 2 School of Social Work, University of British Columbia - Okanagan, 3333 University Way, Kelowna, BC, V1V 1V7, Canada; 3 BC Mental Health & Addiction Services, Provincial Health Services Authority, 70 Colony Farm Road, Port Coquitlam, BC, V3C 5X9, Canada; 4 Faculty of Life and Social Sciences, Swinburne University of Technology, PO Box 218, John St. Hawthorn, 3122, Australia; 5 University of Toronto and the Canadian Network for Mood and Anxiety Treatments, Toronto Western Hospital, Main Pavillion, 9th Floor Room 324, 399 Bathurst St., Toronto, ON, M5T 2S8, Canada; 6 Faculty of Education, University of British Columbia, Scarfe Building, Room 2522-2125 Main Mall, Vancouver, BC, V6T 1Z4, Canada; 7 Department of Psychiatry, University of British Columbia, 2255 Wesbrook Mall, Vancouver, BC, V6T 2A1, Canada

**Keywords:** Bipolar disorder, Interdisciplinary, Community-based participatory research, Knowledge translation

## Abstract

The Collaborative RESearch team to study psychosocial factors in bipolar disorder (CREST.BD) is a multidisciplinary, cross-sectoral network dedicated to both fundamental research and knowledge exchange on bipolar disorder (BD). The core mission of the network is to advance the science and understanding of psychological and social issues associated with BD, improve the care and wellness of people living with BD, and strengthen services and supports for these individuals. CREST.BD bridges traditional and newer research approaches, particularly embracing community-based participatory research (CBPR) methods. Membership of CREST is broad, including academic researchers, people with BD, their family members and supports, and a variety of health care providers. Here, we describe the origins, evolution, approach to planning and evaluation and future vision for our network within the landscape of CBPR and integrated knowledge translation (KT), and explore the keys and challenges to success we have encountered working within this framework.

## Background

“I think it can be difficult for people to fully understand what it is like to experience the dizzying swings from elation to crushing depression inherent in bipolar disorder. Grandiosity and psychosis are akin to possession by an alien force; briefly exhilarating, then terrifying, exhausting and confusing. The aftermath - when you wake up with your whole life in shambles - is possibly the worst of all. Day to day we live with the challenges of grinding self-doubt, irritability, insomnia, anxiety, agitation, self-stigma, social isolation and disability. Bipolar disorder can be the cause of tremendous suffering. Yet there are those of us who live well. Its presence can confer a richness of experience and a desire to soar that many of us parlay into lives of creative and humanistic contribution.”

As team member Sara Lapsley describes in this opening quote, bipolar disorder (BD) is a potentially serious condition characterized by extreme mood states of mania and depression, as well as chronic subclinical mood dysfunction [1]. The condition is more common than previously thought: classical BD type I affects about 1% of the population, but over 6% may fall on the bipolar spectrum [2]. Over half a million Canadians are estimated to experience the condition. Even though BD can be associated with significant strengths (e.g. [3]), its net functional impairment ranks it as the 6th leading cause of disability worldwide [4]. Further, hospitalization rates for BD are increasing among young adults [5] and it has been estimated to be responsible for half of inpatient care costs [6,7], making it a serious public health concern. People with BD must also contend with the effects of stigma resulting from negative social reactions to their illness [8].

Historically, research into BD has been conducted within a biomedical framework, with the bulk examining the biological causes and consequences of the condition, and pharmacological approaches to treatment. Indeed, pharmacology forms the bedrock of treatment for many people who live with BD [9]; however, outcomes in BD vary widely. Even optimal pharmacology fails to ward off mood episodes in some people, and rates of relapse and hospitalization are high. For example, one longitudinal study has shown that 37% of patients taking mood stabilizing medications will relapse back into an episode of depression or mania within one year, 60% in two years, and 73% in five or more years [10]. Further, psychosocial functioning and quality of life (QoL) can remain compromised between mood episodes [11-13].

Although this evidence paints a bleak picture, people with BD can live well, and experience optimal QoL [14,15]. In recent years, we have seen a changing zeitgeist in BD research [16]. Mirroring developments which occurred in schizophrenia research a decade earlier [17], comprehensive biopsychosocial models of BD have now been proposed [18-20], and a number of adjunctive psychosocial treatments found effective [21]. The groundswell of research around constructs such as recovery [22], QoL [23], wellness [15,24] and positive functioning [25] are further evidence of this shifting focus. However, research activities in this area have been fragmented.

## Case description

In this article, we provide a Case Study of the establishment of a unique network centred in Canada, the Collaborative RESearch Team to study psychosocial issues in Bipolar Disorder (CREST.BD). The overarching mission of CREST.BD has been to advance the science, understanding and application of psychosocial issues associated with BD, improve the care and wellness of people living with BD, and strengthen services and supports for these individuals. The article begins by describing the evolution and current status of the network, including illustrative examples of mental health research and promotion the network has undertaken. The values guiding the practices of CREST.BD, including our commitment to community based participatory research (CBPR) principles are introduced. We then describe our approach to strategic planning and evaluation, and explore factors that have either promoted or hindered the network’s success. We conclude with reflections on the potential value this kind of novel approach adds in terms of the quality and impact of mental health research and promotion.

### Origins of CREST.BD

This collaborative team originated with a PhD-psychologist (Erin Michalak) working to develop a program of research into the psychosocial facets of BD. Her early qualitative work examining the QoL construct [26] indicated that myriad psychosocial factors affect outcomes in people with BD, and that many of these factors (e.g., stigma, spirituality, social support, identity) do not fall within the rubric of the biomedical model.

Recognizing the potential utility of a multidisciplinary, collaborative approach, a one-year ‘team planning’ grant from the British Columbian health funding agency was secured to form a provincially-focused team. From the outset, a set of core values emphasizing authentic collaboration and diversity of expertise were explicitly adopted. On these grounds, it was important to expand the team beyond researchers to include clinicians working on the front lines of clinical care. It also entailed valuing the powerful ‘lived experience’ expertise possessed by people with BD, their family members and supports. The success of CREST.BD would in part be determined by ensuring that the range of founding team members endorsed similar values.

A cadre of founding members with expertise across the various psychosocial aspects of BD (spanning the disciplines of psychology, psychiatry, social work and occupational therapy) were recruited. Individuals with expertise across both acute treatment and health promotion, theoretical models, treatment models and regional and cultural divides were invited, as well as people with experience in network development. Some CREST.BD members were international experts in BD research; others were selected for their expertise in complementary fields.

With a founding team in place, the next task was to identify research priorities. First, supplementary funds were secured from the Canadian Institutes of Health Research (CIHR) to conduct a Community Engagement Day (CED, see Table 1). Second, findings from the engagement day were fed into the subsequent team meeting, during which priority areas for research were identified (see Table 1). Third, further work (e.g., literature searches, further consultations, networking) was conducted to select initial research foci (specifically, research designed to explore the QoL construct in BD and psychosocial treatment intervention studies). At the same time, gaps in the team’s expertise were identified and addressed.

**Table 1:** Community engagement day ‘Psychosocial issues in BD: setting the CREST.BD agenda

	
Held in a historic building on UBC campus in Vancouver in the spring of 2008, CREST.BD’s first (of what were to become annual) CED entitled **‘**Psychosocial issues in BD: setting the CREST.BD agenda’ was designed to exchange knowledge with community members and key stakeholders about priorities for psychosocial/BD research. The event, co-chaired by the team leader and a team member with lived experience, was attended by 48 people: 30 community members (people with BD, family members, health care providers and community agency representatives) and 18 CREST.BD members. A variety of methods (interactive presentations, small group breakout sessions and brainstorming sessions) were used to garner input; QoL, wellness and stigma were identified as key research priorities for the team.	To maximise KT, the CED and subsequent team meeting were held back-to-back, with core team members attending both events. The meeting involved 30 participants, representing provincial, national and international expertise in BD research and clinical care. Prior to the meeting, a quantitative email exercise was performed to produce an initial list of potential BD/psychosocial research areas. Specifically, team members participated in a group brainstorming exercise to generate possible research foci, the results of the exercise were collated and participants were then asked to rank each foci on the basis of: i) perceived importance; ii) personal interest and expertise; and; iii) capacity to collaborate. In this facilitated group environment, time was also dedicated to exploring each of the research topics, in the context of the findings from the engagement day, in addition to exploration of the team’s mission and core values.

In summary, this one-year team planning phase allowed CREST.BD to begin the process of: coalescing and establishing a collective identity, exploring core values and mission, identifying priorities for research and gaps in the team - all valuable factors for supporting successful team functioning [27].

### Evolution of CREST.BD

Although multidisciplinary, collaborative approaches are increasingly acknowledged as necessary to address some of the most urgent contemporary health challenges [27,28], CREST.BD, like many emerging teams [29], struggled in the absence of administrative or infrastructure resources necessary to bring these collaborations to fruition. Modest environmental support was provided by the primary host institution, the University of British Columbia Department of Psychiatry, while entrepreneurial partnerships with other networks such as the Canadian Network for Mood and Anxiety Treatments (CANMAT) enhanced the visibility and credibility of CREST.BD. In addition, the team acquired small grants (such as CIHR meeting and dissemination grants and funding from individual members’ own institutions), and continued to build community capacity. The team hosted an annual CED, the topic of which was decided by community priorities: namely, stigma and discrimination (2009); recovery (2010), and creativity (2011). A novel feature of these events was the embedding of a formal research project (see Table 2).

**Table 2:** 2011 Community engagement events

	
Based on feedback from prior community consultations, CREST.BD developed a series of inter-related events for 2011 targeted towards people with BD who self-identified as creative. The first event was a public screening of three documentaries (two by local B.C. filmmakers living with BD), which explored understandings of the role of creativity in BD. A post-screening discussion with one of the filmmakers allowed for greater audience interaction with the issues and themes that emerged from the films. The second event represented the team’s third annual CED, entitled: ‘Touched with Fire or Burnt Out?’: Igniting a dialogue. During the day-long event, 22 participants shared experiences during focus groups designed to explore the factors which encourage or challenge the expression of creativity. Ten team members were in attendance, several of whom presented recent team research on the link between BD and creativity. Graphic facilitation methods [30] were used to capture the themes from the day and additional action-oriented insights from participants.	The final event was held later the same day, where participants and the general public were invited to: ‘The Creative Life: A Night of Music celebrating BD’ held at an arts-focused, community venue. A mural, developed through the graphic facilitation process, was displayed, and served as a focal point for further engagement. The main feature was a live band fronted by a team member with BD, local musicians and team members, who performed popular songs by artists with BD. Each of the inter-related events was co-facilitated by the team leader and a team member living with BD, each with their own unique expertise in the creative arts. Over 250 community members and 20 team members participated. The combined events increased the team’s visibility in the BD community, widened community and health care provider networks, and encouraged community members and wider audiences to engage in this new area of research.

With very limited resources, CREST.BD found ways to develop while still making substantial contributions to the BD research and KT community. Standard metrics of team productivity, such as leveraging of funds and publications, demonstrated a high level of output: With $50,000 in baseline infrastructure investment, the team leveraged $350,000 in research funding between 2007–2010 and produced 16 publications. The team garnered significant media coverage (such as an article in a national newspaper, see http://www.crestbd.ca), recruited over 350 people with BD or their family members to join their ‘Community Consultation Group’ and recruited numerous trainees and peer-researchers (knowledge users collaborating in research). This being said, it took enormous effort and goodwill on the part of the team members to maintain the group in the absence of core infrastructure funding. This effort paid off in 2009, when two of the members co-wrote a grant including 7 additional team members that was ranked top in CIHR’s ‘Knowledge to Action’ competition, securing $200,000 over two years to support KT methods research (see Tables 3 and 4). Critically, this funding allowed the team to begin to establish many of the components necessary for growth, such as the hiring of a KT manager and establishment of a Community Advisory Group (see Table 5). The KT manager was responsible for maintaining partnerships with community members and stakeholders, while the Community Advisory Group, consisting of individuals with lived experience BD, clinicians and organizational representatives, was responsible for providing advice regarding the team’s research and KT activities.

**Table 3:** Health promotion case example 1: Self-management strategies

Self-management strategies	It is now widely recognized that self-management strategies (the decisions and actions an individual takes to cope with or improve their health) are critical for living well with chronic mental health conditions (e.g., [32]). Indeed, Accreditation Canada’s ‘Standard’s for Mental Health Services’ [33] require support for self-management and it is likely that a chronic care model rather than a treatment model is optimal for a waxing and waning condition such as BD [21,34].
	CREST.BD has generated knowledge [8,15,24] on effective self-management strategies for people living well (with BD, identifying five key issues of importance:
	1) Self-management strategies;
	2) Sense of self/identity;
	3) Social support;
	4) Personal growth; and
	5) Addressing internalized-stigma.
	As one component of its CIHR Knowledge to Action grant, the network has worked with peer-researchers to co-produce a series of general audience summaries and workshop modules describing these research findings to disseminate nationally.

**Table 4:** Health promotion example 2: Tackling stigma through theatre

	
One contributing factor to disability and poor QoL in people with mental illness is the degree of stigma they experience [26,43,44]. It is now well-recognized that the stigmatization of mental illness leads some individuals to avoid or discontinue treatment for their mental health problems [45]. CREST.BD has generated knowledge relating to stigma in BD from several research projects, demonstrating, for example, that ‘moving beyond internalized stigma’ (a progression from a state of self-stigmatization to one where society’s stigma toward people with mental illness is no longer internalized [8] is, an important facet of living well with BD [8,46].To share knowledge about stigma in BD with both people with BD and their health care providers, one component of the team’s CIHR KTA grant involves team member Victoria Maxwell (an actress and mental health educator who lives with BD) performing a new one-woman theatrical performance entitled ‘That’s Just Crazy Talk’ commissioned as part of the grant. Although drama has been used with success elsewhere to target mental illness stigma awareness [47,48], this is the first example of such an initiative in the BD field. CREST.BD launched the play in Vancouver and Toronto in July of 2011. Health care providers and people with BD were invited to participate in the research component of the activity, which involved pre- and post-performance questionnaires. The general public was also invited to attend.	The two performances attracted 65 health care providers, 54 people with lived experience, 3 individuals who identified as both, and over 100 additional audience members.In ‘That’s Just Crazy Talk,’ dramatic narrative is used to convey the corollaries of decades of personal and familial mental illness, effectively translating the narrator’s personal experiences of both external and internalized stigma into a vivid, often humorous and sometimes troubled, portrait of life lived with BD. The narrator addresses her family’s profound experiences of mental health stigma and her attempts to come to terms with the implications of her chronic and complex illness. The audience’s intimacy with the narrative is underscored by the community-accessible locations where the play is held, the small audience size (approximately 140 participants per show) and a post-performance question and answer period where Victoria’s ‘character’ is cemented in reality as individuals engage with her present and vibrant self. The lived-experience aspect of the performance is unique in this genre of theatre, and is viewed by the team as a key dimension for using this approach to address stigma. Further performances of ‘That’s Just Crazy Talk’ have been planned in Canada and internationally, and a DVD of the performance has been produced.

**Table 5:** **Key principles of CBPR as outlined by Israel et al. **[38]** [46: 178–180]**

	
1.	Recognizes community as a unit of identity central to CBPR;
2.	Builds on strengths and resources within the community;
3.	Facilitates collaborative partnerships in all phases of the research (e.g., shared control and equitable participation);
4.	Integrates knowledge and action for mutual benefit of all partners;
5.	Promotes a co-learning and empowering process that attends to social inequalities;
6.	Involves a cyclical and iterative process;
7.	Addresses health from both positive and ecological perspectives – attending to biomedical, social, economic, cultural, historical, and political factors as determinants of health and disease/illness; and,
8.	Disseminates findings and knowledge gained to all partners.

### Current status of CREST.BD

During its formative years, CREST.BD primarily operated as a regionally focused team. In 2011, the team was successful in securing the top-ranked application in the inaugural CIHR Network Catalyst competition, securing 3 years of infrastructure funding (~$600,000 with over $400,000 of in-kind support) to support growth and evolution into a national network specializing in BD research and KT. The team’s core mandate is to focus on research, clinical practice, and social change informed by a focus on psychosocial factors in BD and a commitment to CBPR. A relatively recent orientation to research, CBPR in this context refers to research in which individuals with BD and their family members, and other key community stakeholders (e.g., health care providers), are active participants in research and KT.

The large Network Catalyst grant supported establishment of a core staff team under the direction of the Network Leader. In particular, funds were used to continue the KT manager position and employ a network manager. Both positions play a key role in supporting the development and implementation of a strategic plan for the network. Specifically, the network manager role supports the strategic planning process by facilitating stakeholder consultation, and implementing strategic initiatives. The KT manager plays a critical role in conceptualizing and implementing the network’s strategic directions for KT initiatives, development and maintenance of KT and communication platforms.

Although there are other excellent teams internationally specializing in BD research (e.g., [31]), CREST.BD is unique in its multidisciplinary configuration; it now includes team members from a diverse range of disciplines (i.e. psychology, psychiatry, criminology, nursing, social work, gerontology, occupational therapy and genetic counseling), expertise in a breadth of research methods and wide ranging fields of BD specialization (i.e. stigma, psychosocial treatments, advocacy, advanced statistics and psychometrics, assessment, cognition, spirituality, coping, novel technologies, indigenous, youth and aging populations). Further, it is the only cross-sectoral BD network - integrating ‘lived experience’ or community expertise, the public health sector, community agencies and academia - to specialize in research committed to integrated KT. A further strength of CREST.BD is its significant international linkages, particularly to the US, Australia and the UK.

### Community-based participatory research

CREST.BD adheres to the principles of CBPR (see Table 5). Driven by mandates to produce practical and applied research that facilitates health promotion and addresses health disparities, researchers are increasingly employing CBPR approaches to address complex health and social problems [35-38]. At its core, CBPR can be defined as scientific inquiry that is conducted as a partnership between researchers and community members affected by a particular health condition, disability or issue. It is characterized by substantial community engagement in the development and implementation of the research including formulating conclusions and communicating results [39]. It is particularly suitable for working with socially disadvantaged communities, whose members experience limited access to resources and who are disproportionately affected by health disparities. The emphasis is on integrating the generation of knowledge into strategies that contribute to provide community and social change [38], with research being understood not only as a process of creating knowledge, but simultaneously, as education and empowerment, and of mobilization for action [40]. Community-based or participatory approaches represent a philosophy of engagement [41] rather than a discrete research method - researchers employing CBPR can draw on any number of qualitative and/or quantitative methods to achieve the identified research goals [42].

In the next section, we present what we understand to be the keys and challenges to success in the establishment of CREST.BD, focusing on six areas: 1) Participatory leadership and power-sharing, 2) Establishing and maintaining identity, 3) Attention to communication, 4) Optimal use of resources, 5) Flexibility vs. sustainability, and 6) Optimizing and Evaluating Success and Impacts. Although these are described as discrete facets, the concepts intersect, complement and overlap.

Consistent with our emphasis on CBPR a premise of CREST.BD is that research should not be separated from its implementation, consequently, KT has become a central modus operandi of the network [49]. We understand KT as: “a dynamic and iterative process that includes, synthesis, dissemination, exchange and ethically sound application of knowledge” [50]. An integrated model of KT is heavily focused on the practices of knowledge exchange; that is, the sharing of knowledge between and across research partners and knowledge users. This approach emphasizes collaboration with those directly involved and who have personal knowledge and experiences of BD, under the assumption that this will maximize the likelihood of delivering relevant, pragmatic and effective interventions [51]. In a parallel fashion, providing knowledge and support relevant to learners – a primary tenet of effective continuing education - is done in an explicit way that changes health care delivery at the level of the health care provider. We believe that such an approach is necessary to drive policy reforms which can improve access to resources and ultimately deliver better mental health outcomes for individuals living with BD and their family members.

### Keys and challenges to success

#### Participatory leadership and power-sharing

A participatory leadership approach is one which prioritizes respect and engagement to harness diversity, build community, and create shared responsibility for action [52,53]. This commitment to egalitarian and participatory decision making contrasts with hierarchical leadership, and aligns well with CREST.BD’s commitment to CBPR. Participatory leadership is promoted by commitment to authentic, reciprocal research partnerships, patience, flexibility in research designs and transparent communication of anticipated and actual research outcomes [36,42,54]. Integral to the success of participatory leadership is the inclusion of members a diversity of lived and learned experiences which allows for generous sharing of “what works” throughout the group (as opposed to directive leadership) as well as a unique training environment.Power-sharing is integral to participatory team work. Governance structures are one avenue for power-sharing, therefore CREST.BD has established community structures such as a Community Advisory Group, Community Consultation Group and most recently, a National Advisory Group (see Tables 6 and 7). Power-sharing has been further supported by including community members as co-investigators or knowledge users on funding applications (where a proportion of the budget is diverted to the community group) and fostering transparent dialogue about the risks and benefits, goals, and anticipated outcomes of research [54]. Finally, a key to success in our partnerships with community members and stakeholders has been the identification of research priorities important to them (see Table 1). As CREST.BD has grown, linkages to other organizations have been strengthened. Most impressively, solid attention to the Community Advisory Group has led to more formal alliances and joint grant submissions with the Mood Disorders Association of BC and the Mood Disorders Association of Ontario. Attention to the linkages with service providers has led to a “Principal Knowledge User” [55] partnership with CANMAT, which allows for identification of needs of health care providers as well as implementation of research findings in the health care sector.

**Table 6:** CREST.BD governance and community partnerships

	
Governance	CREST.BD has established itself based on its commitment to authentic engagement with community members. The CREST.BD ‘Community Advisory Group’ (CAG) consists of 8 members representing: people living with BD, BD health care providers or organisation representatives. The broad objective of the CAG is to provide advice and feedback on CREST.BD’s on-going research and KT activities.
	More specifically, the CAG:
	1. Act as a resource to CREST.BD in terms of planning, implementation, distribution and evaluation of research studies and KT;
	2. Helps to generate solutions to barriers within the research and KT initiatives.
	3. It has also played a key role in optimizing networking opportunities with the wider BD community;
	4. Function as a communications vehicle to the BD community on the work and plans of CREST.BD;
	5. Problem-solve barriers and solutions within the team’s research and KT initiatives.
	Although the CAG was provincially-focused and populated it is now being reconfigured for a national network. CREST.BD also established community structures at a provincial level that have been expanded to a national level. For example, the team maintains a Community Consultation Group of over 350 people living with BD, family member, supports and health care providers. The consultation group has been engaged with CREST.BD activities in a variety of ways, including, developing a new QoL scale, designing the team logo, designing and populating the team website and social media platforms, disseminating information about research and KT activities. In line with CBPR principles, the team’s relationship with the CCG is bi-directional; CCG members provide the team with insights, information and knowledge and CREST.BD offers the same in the form of regular newsletters, outreach, access to research findings and other KT.
Community partnerships	In addition to working closely with community members, CREST.BD has been active in developing and maintaining partnerships and alliances with key stakeholder organizations. For example, CREST.BD has a successful partnership with CANMAT (www.canmat.org), an academic not-for-profit research organisation linking health care professionals from across Canada who have a special interest in mood and anxiety disorders. CANMAT, with its 15 year history, has powerful visibility and credibility to clinicians and policy-makers across Canada. Since CANMAT focuses more on biological research and treatments, it too can benefit from CREST.BD by demonstrating a broader commitment to holistic treatment via enhanced attention to psychosocial treatments. CANMAT also has significant operational experience in creating and disseminating multiple educational tools and events.
	More recently, CREST.BD has established a National Advisory Group (NAG) which has a wider remit (e.g., identification of strategic directions and new national-level partnerships) than the more project-focused CAG. The NAG has national and policy-level representation (e.g., Mental Health Commission of Canada, Canadian Health Services Research Foundation, Canadian Mental Health Association, the Mood Disorders Society of Canada.

**Table 7.** CREST.BD communication strategies

	
As with any academic team, we publish our research findings in peer-reviewed journals. However, working in a multidisciplinary environment affords us additional leverage in terms of capitalizing on our research findings. For example, we published the primary findings from our self-management strategies for BD study in a top-ranked mood disorders journal [15]. Additional team members then went on to explore the clinical implications of the findings [24], perform an in-depth analysis of the role of stigma [8] and replicate the study in a New Zealand population [61]. We also endeavour to transform the findings from our academic publications into other more accessible formats (e.g., content for newsletters, magazine articles), media (e.g., radio or television segments, public discussion forums), or materials that give voice to lived experience perspectives. In 2012 the network will be the subject of an in-depth Canadian Broadcasting Company (CBC) radio piece, the development of which was supported by a competitive CIHR Health Journalism Award.	Underpinning our communications are three web platforms: a team website (www.crestbd.ca), Twitter (www.twitter.com/CREST_BD) and Facebook page (www.facebook.com/CRESTBDBipolarResearch), which continue to demonstrate sustained growth in visitor traffic. For instance, from January to April 2011, the website welcomed 1,320 new visitors and 714 returning visitors; between January and April 2012 this had increased to 2027 new visitors and 1028 returning visitors. The visitors in this first quarter of 2012 also demonstrated increased engagement; spending, on average, more time on the website. Our CREST.BD Facebook page, launched in February 2011, received over 6554 post-views in June 2012, an increase from 1844 views from June 2011. On Twitter we now host over 130 followers including international researchers, journalists, mental health organizations and KT specialists, and @CREST_BD is featured on several subscribed Twitter lists. The website has a number of interesting features, including scientific publications and presentations tagged by theme, *Bipolar Currents -* general audience snapshots of our research findings, pages dedicated to sharing our major research projects and products (e.g. That’s Just Crazy Talk, Quality of Life in Bipolar Disorder Scale), embedded YouTube videos and Slideshare presentations from our branded CREST.BD channels, and a live Twitter feed representing further modes of KT. With multiple social media and website updates occurring each week, we expect a continued increase in viewership of this multi-modal, web-based KT infrastructure. Further, this KT infrastructure has the potential to facilitate our outreach into rural and remote communities.
In some CREST.BD activities, our research and communication goals are closely intertwined. For example, our ‘Recovery Narratives’ project pairs up a clinician team member with a person with BD who wishes to share their story of recovery from BD. The primary research objective of the study is to explore the impact of clinician-guided recovery narratives on various outcomes, measured quantitatively and qualitatively. However, there are also more rapid KT outputs from the study as the written recovery narratives are made available via the team website to the wider community.	

The utopian view of participatory team work is that we are all working together to achieve shared goals (see Table 5). In reality, collaborations between researchers and community members can be strained by a lack of mutual understanding of each other’s agendas, expectations and needs. CREST.BD’s knowledge users include researchers, people with lived experience, health care providers, community agencies and advocacy groups; at times, these stakeholders have different agendas. Our experience has been that people with BD and their family members place high value on advocating for social change; whereas researchers value the team’s research activities. Whilst the two goals are not mutually exclusive, they are not always fully integrated; CREST.BD’s primary remit is research and KT, and at times strategic leadership has been required to keep the team’s development and evolution into a network on track.

#### Establishing and maintaining identity

At the outset, a priority for CREST.BD was to establish a solid identity based on scientific reputation, niche expertise, credibility, visibility, and a shared sense of purpose. Visioning exercises around team identity were conducted at annual team meetings. Additionally, branding facilitators (e.g., team logo and branded website, Facebook and YouTube sites) were produced. This collective identity has been paramount in leveraging funds and other returns, and has fostered the shared sense of values, vision, and purpose by team members [56]. Having achieved this intra-team common ground and recognition as a leading research group in a niche area of BD research, CREST.BD’s identity provides a solid foundation from which to attract new partners and collaborators [27].

**Figure 1:**
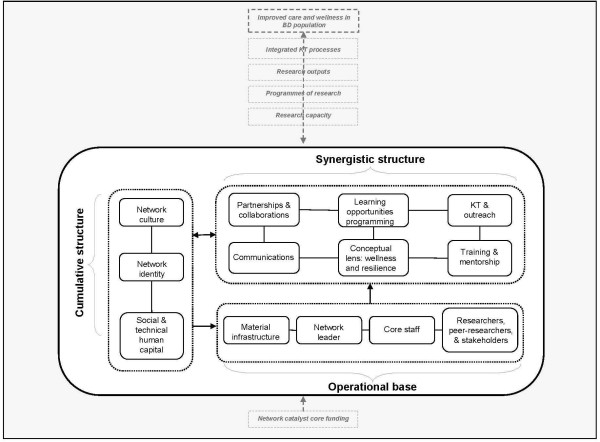
Diagram of network configuration.

One potential threat to identity is ‘mission drift’, as exemplified by seeking funds outside of the network’s mandate. Mission drift can be minimized by transparent accountability to governance systems and open communication with the wider community. In a relatively small organization, mission drift can also result from membership decisions - the core mandate is not advanced by accepting new members who may increase the profile or fundability of the group, but who do not share the group’s culture. Culture encompasses the values and norms that guide how the network’s research and KT are undertaken, and is a force that shapes collaborative behaviour and functioning [27]. (See Figure 1: *Diagram of Network Configuration* (Derived from [27]).

One of our primary conceptual lenses is an orientation towards wellness or resiliency, instead of dysfunction and pathology. At the same time, there is open discussion of conflicting agendas; acute care priorities impel certain research directions while the focus on wellness places greater emphasis on others. A project involving the theatrical presentation of the impact of BD on one individual provides an illustrative example (see Table 4). This theatre-based intervention vividly demonstrated difficult encounters with the traditional health care system. During the theatrical performance, a psychiatrist team member who co-wrote the grant that funded the project was on-call providing the type of hospital-based treatment that the performance gently parodies. The exigencies of acute care encourage some team members to advocate for research relevant to acute care settings, while others continue to advocate for the wellness perspective. Membership from both camps is important to the ultimate relevance of CREST.BD.

#### Attention to communication

Well-honed, tailored communication strategies are a central component of effective integrated KT, serving to build community cohesion and support group identity [27]. We have used a blend of, face-to-face, written and virtual interactions including regular e-newsletters a dedicated website, Facebook and Twitter accounts, YouTube channel and (see Table 7). Team members communicate via a members-only project management system where study protocols, budgets, meeting minutes and ethics documents are centrally housed to facilitate transparency and accountability. We have created a database of general audience summaries of our team publications, new research and KT initiatives in order to foster transparency, share knowledge and encourage community engagement.

Although CREST.BD is based on a commitment to authentic engagement and communication, this process has not always been easy. Before the team secured its CIHR KTA funding and associated support for a KT manager, most of the day-to-day communication with community members and partners was handled by the team leader. At times this high level of communication could be intense, especially for an early-career researcher. As CREST.BD develops from a provincially-focused team into a nationally-focused network we will also need to attend to issues such as the engagement of all provinces/territories and strategies for reaching Francophone communities and rural/remote or Northern communities (see External Communications section).

#### Optimal use of resources

Leveraging existing resources has been a key to CREST.BD’s success. We have built capacity by: centralizing resources and reducing redundancy and duplication of efforts; providing stakeholders with access to existing resources through new communication channels; and providing a unique training environment for students, new investigators, clinician-scientists and peer-researchers. We have provided a platform where individuals based in other universities and organizations have an additional venue for visibility and action; this in turn facilitates sharing of resources from those partners, fostering mutual success. As a national network, we have the ability to source resources and knowledge in-house, allow for sharing of facilities and ‘economy of scale’ in expertise, decrease the risk of duplication of resources and to assimilate knowledge from different programs of research (i.e., synthesize research evidence). As part of our goal to leverage existing resources we are conducting a national environmental scan of BD/psychosocial research, KT and treatment programs.

As participatory research tends to be resource and time intensive, optimal use of resources is required [28]. Coordination of research activities including community engagement and obtaining funds to support both community and academic infrastructure can be overwhelming [57]. In addition, key steps of CBPR such as assessing the community’s needs, getting to know the community, and developing strong sustainable ties with partners takes significant time [28,58]. These challenges can result in practices where researchers are accused of adopting a ‘hit and run’ model of consultation, leading to distrust and frustration and undermining core values of CBPR [37,42]. CREST.BD encountered particular issues around the speed with which it was reporting outcomes from its combined research and consultation days (see Table 2). For example, there were tensions inherent in expediently describing the ‘results’ from a community engagement day that incorporated qualitative focus groups requiring in-depth analysis. As one remedy to this, we now produce ‘research snapshots’ that summarize key process or high-level findings in a more expedited manner.

#### Flexibility vs. sustainability

Working within a multidisciplinary team with various forms of expertise means we can be flexible in response to different kinds of funding opportunities (e.g., operating, end-of-grant or integrated KT, KT methods, infrastructure funding). The pre-established working relationships in the team and centralized resources (e.g., KT and network manager and a cadre of trained peer-researchers) ensure that we are nimble in response to funding calls. However, a common challenge in CBPR research and network development alike relates to long-term sustainability [27,28]. CREST.BD was formed on the basis of a one-year funding award, which proved critical for the *process* of conceptualizing the new team. However, decreased government support to the provincial funding agency resulted in the discontinuation of their team funding stream and CREST.BD was without infrastructure funding for a 3-year period. Sustaining networks remains an ongoing dilemma [27], and the network will need to attend to sustainability factors closely. As noted above, in our favour is the network’s inherent flexibility; we will be in a position to react quickly to contextual changes, including societal demands for knowledge and new funding opportunities. Further, we anticipate that our integrated KT framework will ensure that network research priorities will be malleable to new demands for knowledge over time. Finally, the infrastructure and partnerships we have developed are integral assets of the CREST.BD research environment and should create a lasting legacy.

#### Optimizing and evaluating network performance

**Figure 2:**
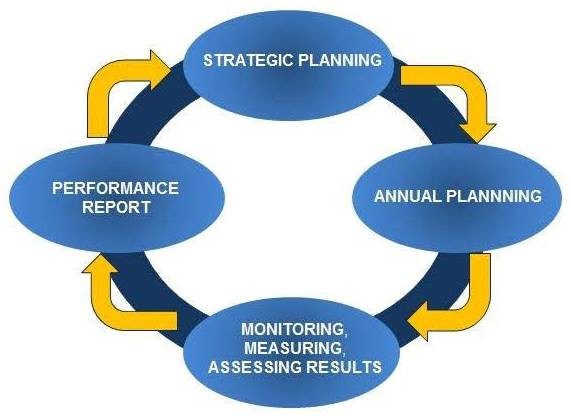
Continuance performance management cycle.

**Figure 3:**
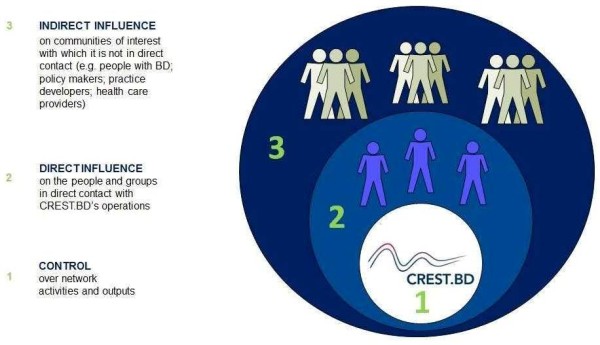
Continuum of control concerning the network’s activities, outputs, and impacts.

CREST.BD has adopted an integrated performance management framework in order to formulate and monitor the strategic planning process for the national network. This framework is comprised of a continuous performance management cycle (see Figure 2: *Continuance Performance Management Cycle*) a logic-based theory of change model (adapted from C. Bennett, 1979 [59]), and a continuum of control concerning the network’s activities, outputs, and impacts (adapted from S. Montague, 2011 [60], see Figure 3: *Continuum of control concerning the network’s activities, outputs, and impacts*). Together, these elements are being used to establish and adapt the strategic goals and directions of the network. In keeping with its core values, CREST.BD is also using participatory processes that prioritize the involvement of network members and stakeholders in shaping the strategic plan.

**Figure 4:**
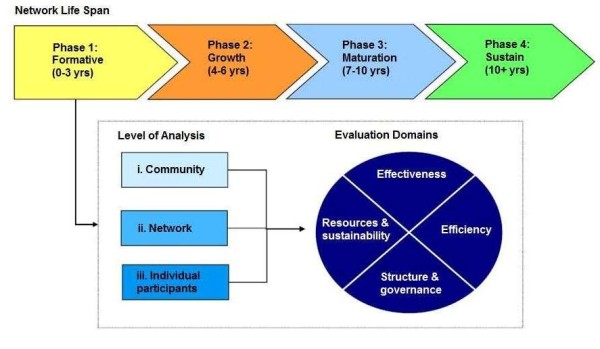
Four key domains for knowledge networks.

In conjunction with this strategic planning process, a range of evaluation approaches are being drawn upon to assess the performance and impact of the CREST.BD network. Our evaluation framework features a logic model outlining the network’s inputs, outputs, and outcomes. In addition, we wanted to ensure that our evaluation framework was aligned with the dynamic, innovative, and non-linear nature of a KT network. Therefore, our framework incorporates the tools of developmental evaluation, which allow for information to be gathered in a way that informs and supports the ongoing development of a network, as opposed to measuring its performance at a single, fixed period of time. Finally, our evaluation is designed to attend to the nuances of a KT network; as such, the performance indicators are tailored to the fact that CREST.BD sits between the formative/start-up and growth phases of a network lifecycle. Moreover, the performance indicators span four key domains that are considered important for evaluating knowledge networks, including resources and sustainability, structure and governance, efficiency, and effectiveness (see Figure 4: *Four key domains for knowledge networks*, [62]).

## Conclusions

We believe that the model epitomized by CREST.BD has the potential to pay real dividends in terms of improving the quality and effectiveness of mental health research and promotion. We believe our CBPR orientation can: support the development of research questions that reflect health issues of priority to community members [42]; improve research recruitment and retention, and the quality of research data [42]; and increase the relevance and pragmatism of mental health interventions [42,51]. Furthermore, these methods hold the potential to improve health and wellbeing in the community members, increase health equity and citizenship and mobilize social change for people living with mental illness. Or, as team member Sara Lapsley describes it: 

“CPBR brings the BD community into the academic conversation, empowering those with BD by eliciting expertise accumulated through years of lived experience. Informed by our perspective, researchers can work collaboratively with us to guide research questions and meaningful interpretations of the results. By using innovative KT methods to convey the findings in a way that is relevant to the BD community, CPBR helps spread the message of hope, and bestows on us the gift of engaging in our own recovery”.

Bipolar disorder can impose a tremendous burden on those living with the condition and their family members and does impose a great burden on the Canadian health care system. We have seen a groundswell of research into psychosocial factors and treatments in BD, and there now exists a body of knowledge that, if applied, could make a significant difference to the QoL of people living with BD, their health care providers and the Canadian economy. In this article we have described the origins, evolution and current status of a collaborative network dedicated to researching and exchanging knowledge on psychosocial factors in BD. We hope to have demonstrated some of the added-value that arises by partnering with communities in new ways, both in terms of enhancing research quality and changing the landscape of research and care for people living with mental illness.

## Abbreviations

BD: Bipolar disorder; CAG: Community Advisory Group; CANMAT: Canadian network for mood and anxiety treatments; CBPR: Community based participatory research; CED: Community engagement day; CIHR: Canadian Institutes for Health Research; CREST.BD: Collaborative RESearch team to study psychosocial issues in bipolar disorder; KT: Knowledge translation; QoL: Quality of life.

## Competing interests

The authors declare that they have no competing interests.

The **C**ollaborative **RES**earch **T**eam to study psychosocial issues in **B**ipolar **D**isorder (CREST.BD, http://www.crestbd.ca) is a team of researchers, clinicians, and community members dedicated to developing knowledge on bipolar disorder. The team includes representatives from a variety of health disciplines, including psychology, psychiatry, occupational therapy, nursing, criminology, genetic counselling, and mental health advocacy. A guiding principle of CREST.BD is to foster and promote ‘Community-based Participatory Research’, whereby individuals with BD and their family members are active participants in research and knowledge translation.
